# Risk assessment of thyroid nodules with a multi-instance convolutional neural network

**DOI:** 10.3389/fonc.2025.1608963

**Published:** 2025-07-24

**Authors:** Da Yu, Tingting Song, Yancheng Yu, Hebin Zhang, Feng Gao, Zirong Wang, Jiacheng Wang

**Affiliations:** ^1^ Department of Ultrasound, The Affiliated Hospital of Hangzhou Normal University, Hangzhou, Zhejiang, China; ^2^ Department of Radiology, The Fourth People’s Hospital of Harbin, Harbin, Heilongjiang, China; ^3^ Department of Radiology, The Affiliated Hospital of Hangzhou Normal University, Hangzhou, Zhejiang, China; ^4^ Department of Computer Science, School of Informatics, Xiamen University, Xiamen, China

**Keywords:** multi-instance learning, convolutional neural network, thyroid nodule, ultrasound image feature, thyroid cancer diagnosis

## Abstract

**Objectives:**

Ultrasonography is the primary imaging modality for evaluating thyroid nodules, and artificial intelligence (AI) has advanced the automated diagnosis of thyroid cancer. However, existing AI-assisted methods often suffer from limited diagnostic performance.

**Methods:**

In this study, we propose a novel multi-instance learning (MIL) convolutional neural network (CNN) model tailored for ultrasound-based thyroid cancer diagnosis. The model extracts nodule-level ultrasound features from instance-level images using CNNs, and employs an attention mechanism to assign importance scores and aggregate features across instances. This enables effective feature extraction and localization of key instance features, facilitating risk assessment of thyroid nodules. The dataset consists of ultrasound images from 2000 patients at the Affiliated Hospital of Hangzhou Normal University, collected between 2018 and 2024. The images were divided into training (75%, 1500 patients) and testing (25%, 500 patients) sets. The model's performance was evaluated using metrics, including accuracy, precision, recall, F1-Score, and AUC. To assess the statistical significance of the model’s performance relative to other methods, a paired t-test was conducted based on the prediction results.

**Results:**

The performance of the model developed in this study was evaluated and compared with popular ultrasound image classification models for thyroid nodules. The model outperformed the other two classification models (accuracy 0.8386±0.0334, 0.7999±0.0188, 0.7839±0.0267; precision 35 0.8512±0.0301, 0.9039±0.0154, 0.9267±0.0235; recall0.8427±0.0313, 0.7497±0.0163, 0.6987±0.0249; F1-Score 0.8380±0.0344, 0.8196±0.0178, 0.7967±0.0251; AUC 0.8900±0.0309, 0.8851±0.0124, 0.6340±0.0200), where values are under 95% confidence interval. Statistical analysis showed that the performance differences were statistically significant (p <0.0001).

**Conclusions:**

These results demonstrate the effectiveness and clinical utility of the proposed MIL-CNN framework in non-invasively stratifying thyroid nodule risk, supporting more informed clinical decisions and potentially reducing unnecessary biopsies and surgeries. Codes will be available at GitHub - rrrr-ops/Thyroid-AI.

## 1 Introduction

In 2020, the World Health Organization’s International Agency for Research on Cancer (GLOBOCAN) Global Cancer Statistics showed that thyroid cancer had a worldwide incidence of approximately 586,000 cases, which ranked 9th in incidence, with the highest percentage being papillary thyroid cancer, which accounted for approximately 84% of all thyroid cancers (1, 2). Thyroid cancer may occur in any sex, with women accounting for approximately 75% of all thyroid cancer patients (3). Chinese scholars counted the epidemiological characteristics of 9,662 cases of thyroid cancer from 2014 to 2019 and found that the number of thyroid cancer cases showed an increasing trend year by year, and the growth rate of 2017–2019 was significantly higher than that of the previous three years (4). In 2019, 230,000 new cases and 45,000 deaths were associated with thyroid cancer worldwide, with China accounting for 16.71% and 15.88% of cases, respectively. China has the highest incidence and mortality rates of thyroid cancer worldwide, and the incidence of thyroid cancer in Zhejiang Province has risen sharply (5–7). In parallel with economic development and advancements in early detection, thyroid nodules have drawn increasing clinical attention (8). Thyroid cancer has a significant impact on public health, and despite its relatively low mortality rate, the rising incidence of thyroid cancer has increased the demand for healthcare resources and put tremendous pressure on the public health system. With the aging of the population, the incidence of thyroid cancer is likely to increase further, posing an even greater challenge to the public health system.

The Expert Committee of the Superficial Organs and Vascular Group of the Division of Ultrasound Medicine of the Chinese Medical Association (CMA) formulated the Chinese Guidelines for Ultrasound Malignancy Risk Stratification of Thyroid Nodules (C-TIRADS) in 2020, which is more in line with China’s actual situation (9). This classification system integrates multiple ultrasound features of nodules, such as nodule morphology, margins, internal echogenicity, and calcification, to classify nodules into different categories, from category 1 (no nodule, 0% risk of malignancy) to category 6 (biopsy-confirmed malignancy), with an increased risk of malignancy. The C-TIRADS classification provides critical guidance for clinical decision-making. For nodules classified as category 2 by C-TIRADS (0% malignancy probability), fine-needle aspiration (FNA) biopsy is usually not necessary, whereas for nodules in categories 4B to 5 (10% to >90% malignancy probability), FNA is required for further evaluation (10). The C-TIRADS classification helps clinicians determine which patients are suitable for surgical treatment and which patients provide more conservative treatment options with active monitoring for follow-up or ultrasound-guided thermal ablation (11). Accurate nodule stratification with C-TIRADS facilitates early identification of high-risk nodules and timely therapeutic intervention, thereby improving patient outcomes. In particular, low-risk papillary thyroid microcarcinoma (PTMC) may be managed with active surveillance to avoid overtreatment, whereas high-risk nodules require prompt surgical intervention (12).

C-TIRADS plays an important role in the diagnosis and treatment of thyroid cancer; however, its practical application faces some challenges. First, sample heterogeneity was a key issue. Thyroid nodules exhibit diverse characteristics, including cystic nodules, homogeneous hypoechoic nodules, nodular goiter, calcified nodules, microcalcifications, and diffuse microcalcifications, which can be benign, malignant, or suspected malignant, with varying degrees of severity (13). Since the ultrasound manifestations of thyroid nodules may be similar, and their biological characteristics and treatment options may be very different, C-TIRADS should be able to accurately differentiate between different types of thyroid nodules. Second, the subjectivity of the diagnosis is challenging. The diagnosis of ultrasound images is highly subjective, and there are differences in the interpretation of ultrasound features of thyroid nodules by sonographers with different experiences and inter- and intra-observer variations in the assessment of ultrasound metrics of the nodules, which may affect the accuracy and consistency of the C-TIRADS classification (14, 15). These subjective variations underscore the clinical need for automated diagnostic systems that can provide standardized and reproducible assessments across institutions and operators. In addition, the C-TIRADS provides detailed management recommendations and clinical guidance; however, many thyroid surgeries in China are based on ultrasound diagnostic reports rather than FNA results, sometimes in combination with other relevant clinical bases. Appropriate adjustments are required for practical applications according to regional differences, national characteristics, cultural traditions, and medical systems (9).

Artificial intelligence (AI) has been widely applied to medical tasks including detection, classification, and segmentation (16, 17). In particular, with the rapid advancement of AI technologies, machine learning and deep learning have become promising approaches for the automatic classification of thyroid nodules, and are now extensively used in ultrasound imaging, fine-needle aspiration (FNA) biopsy, and thyroid surgery (18). The general steps of AI for the diagnosis of thyroid nodules include: (i) modeling optimized preprocessing of ultrasound images and selecting the region of interest from them, (ii) applying feature extraction methods for training to classify thyroid nodules, and (iii) diagnosis of benign or malignant nodules. AI is increasingly used in the field of ultrasound with the continuous progress of algorithm iterations and accumulation of clinical data (19–21). Compared to traditional thyroid cancer diagnostic methods, such as FNA biopsy, AI promises to provide a non-invasive alternative for classifying thyroid cancers with higher accuracy by analyzing their histopathological features, clinical manifestations, and prognosis. For example, Li et al. collected more than 40,000 patients with thyroid nodules and jointly used two models commonly used in deep learning, ResNet and DarkNet, to differentiate between benign and malignant thyroid nodules, and obtained an AUC of more than 0.9 (22).These findings suggest that AI has the potential for cross-center use in the diagnosis of thyroid nodules. Peng et al. developed an AI deep learning model (ThyNet) to differentiate between thyroid cancer and benign thyroid nodules (23), and the results showed that the AUC of the features obtained by the ThyNet model-assisted diagnosis was 0.922, which is significantly higher than that of the physician’s 0.839. The percentage of patients with fine-needle aspiration biopsies decreased from 61.9% to 35.2% and the percentage of missed thyroid cancer lesions decreased from 18.9% to 17.0% when using the ThyNet-assisted system. The ThyNet model showed better diagnostic specificity and accuracy than specialized physicians.

However, traditional AI-assisted thyroid cancer diagnostic techniques have some limitations that need to be addressed. First, the diagnostic technique based on a single ultrasound image is limited by the one-sidedness of the information, which is easily affected by a variety of factors, such as imaging quality, patient position, and physician experience, leading to misdiagnosis or missed diagnosis (23–25). Second, traditional techniques are difficult to apply to highly heterogeneous ultrasound image features presented by different patients or even the same patient in different disease courses, making it difficult to cope with complex and changing conditions. In addition, annotation based on a single image not only increases the burden on medical resources but also easily leads to inconsistent diagnostic results due to physician qualifications and regional differences (26). Therefore, these models face challenges in clinical applications because they lack the ability to integrate information regarding the patient as a whole.

In recent years, multiple-instance learning (MIL) techniques have provided new ideas for solving these problems (27–29). MIL is a subfield of machine learning, a paradigm that learns data relationships from a set of instances and bags, and by virtue of the unique structure of bag-instances, is able to not only solve the problem of computational resources in large-scale images. Existing MIL methods have made significant progress in the field of pathology images, achieving substantial improvements in computational efficiency, instance feature extraction, and instance aggregation mechanisms, making them an important for the pathological image analysis. However, the application of MIL in ultrasonography remains to be explored.

Unlike conventional deep learning methods based on single-frame ultrasound images, our model uses a multiple-instance learning framework to aggregate information across frames, enhancing robustness and aligning more closely with clinical practice. The model can receive a collection of patient ultrasound examination video frames as input, and by synthesizing and analyzing the information in these image frames, it can comprehensively evaluate the patient’s condition and provide accurate thyroid cancer diagnosis results. In addition, we compared the feature extraction capabilities of multiple classification networks in the ultrasound diagnosis of thyroid cancer to further improve diagnostic accuracy and model robustness. We further analyzed the interpretation of the model predictions and compared the computational costs, which has proven that our model has potential to be applied in practice.

## 2 Materials and methods

### 2.1 Thyroid nodules risk prediction model based on multi- instance learning

#### 2.1.1 Multi-instance learning framework and problem definition

MIL, as a weakly supervised learning method, is particularly suitable for dealing with the situation where there are multiple relevant instances in the image data but only the overall label is known. Traditional single-instance learning assumes that each training sample has an explicit label, whereas multi-instance learning assumes that each training sample consists of multiple instances, and only the overall label is known. MIL requires that the data be represented in a package-instance structure; when there are positive instances within the package, the whole package is labeled as a positive package; otherwise, the package and all its instances are negative.

The rationale for selecting MIL in this study, as opposed to other algorithms, is driven by the objective of performing thyroid nodule risk assessment based on the patient’s comprehensive information. Specifically, the ultrasound examination process of a patient can be collected as a set of images, referred to as a bag, where each image is considered an instance. The number of instances per patient can vary flexibly according to the actual examination conditions, without the need for consistency. The attention mechanism in the MIL framework is capable of handling dynamically changing instance numbers, effectively adapting to this variable-length input structure. The risk reflected in a single image may not always correspond to the patient’s actual risk level. For instance, certain images from high-risk patients may not directly indicate any evident risk. Given the multifaceted nature of thyroid nodule risk assessment, relying solely on individual images may lead to inaccurate predictions. In this context, MIL is employed to analyze all images of a patient collectively, synthesizing the information from each instance to yield a more accurate and holistic risk assessment (30, 31). Therefore, the architecture of MIL makes it relevant to the context of medical image analysis and risk assessment.

In the MIL model, the instance feature extraction network and instance feature aggregation mechanism are two core components. Instance feature extraction networks are typically designed based on CNN and transformers. In this study, we adopt a CNN-based backbone network. Compared to transformer-based models, CNNs offer two key advantages: (1) they are more effective at capturing local spatial features through multi-layer convolution and pooling operations, which is particularly important for texture-rich ultrasound images (32); and (2) their relatively simple architecture and fewer parameters help reduce the risk of overfitting—a critical consideration given the size of our dataset (2,000 patients, including 1,500 in the training set). The learning objective of these networks is to extract the representative features from each instance. By contrast, instance feature aggregation mechanisms are used to integrate features from multiple instances into package-level features. Commonly used aggregation methods include global average pooling (GAP), maximum pooling (MP), and attention mechanisms. The attention mechanism plays an important role in this study as it can guide the model to focus on key image segments with diagnostic significance, thus improving the accuracy of diagnosis and interpretability of the model. In addition, the feature fusion strategy is optimized to enhance the accuracy of bag-level prediction using methods such as multi-layer perceptron (MLP) to effectively fuse instance features.

In this paper, we propose a thyroid nodules risk prediction algorithm based on multi-instance learning, which aims to reduce reliance on image-level labels and focuses on the extraction and analysis of key instance features. In this algorithm, the set of ultrasound images of each patient was considered a package, whereas each image in the image set was treated as an instance, and the overall risk label of the package was determined based on the comprehensive examination results without the need to label each instance individually. For thyroid nodules risk prediction, the model can extract discriminative key representations from ultrasound images using an improved instance feature extraction network based on convolutional neural networks. Subsequently, in the instance feature aggregation mechanism, the model can select the key ultrasound images that match the clinical experience as the basis for judgment and locate the location and extent of the lesions in the key images. During the training process, the MIL model derives bag-level labels by learning the instance-level information, thereby effectively addressing the challenges of data heterogeneity and complexity. Combined with the MIL approach, the model can simultaneously analyze multiple related images of ultrasound species, jointly analyze the patient’s condition, and provide a more accurate diagnosis.

#### 2.1.2 Model architecture

In this study, we constructed a package containing the ultrasound images of each patient’s thyroid ultrasound results. The model designs a VGG13 based instance feature extraction module and implemented the aggregation of instance features using an attention mechanism.

First, in the example feature extraction module, the model employs the VGG13 network pretrained on ImageNet, a large-scale natural image dataset, and is further trained on thyroid ultrasound images. VGG13 is known for its unique convolutional layer stacking structure, which progressively extracts features at different levels in an image through multiple consecutive convolutional and pooling layers. It relies on successive 3×3 convolutional kernels and rectified linear unit activation functions to deepen the network. Each convolutional layer is typically followed by a pooling layer to reduce the spatial resolution of a feature map. To enhance the adaptability of the VGG13 network to thyroid ultrasound images, we introduced various data enhancement techniques, such as random rotation, scaling, and panning, during the training process to improve the generalization ability of the model and enhance its robustness to data noise. The instance feature extraction module extracts the deepest feature maps of VGG13, and after pooling them, the features of each instance are obtained for subsequent attentional learning. After obtaining multiple instance features, it is crucial to know how to aggregate these instance features to obtain patient-level thyroid cancer diagnosis features. To this end, the algorithm refers to the classical attention-based multi-instance learning method AB-MIL (33) which learns the importance of each instance in the final diagnosis through matrix operations and tanh(.) function and assigns different weights to each instance to obtain patient-level feature representations through weighted summation.

Specifically, given a set of instance features $\text{H}=\left[{\text{h}}_{1},{\text{h}}_{2},\ldots ,{\text{h}}_{\text{n}}\right]\in {\mathbb{R}}^{\text{N}\times 512}$ , where N is the number of instances and each ${\text{h}}_{\text{i}}$ is a feature vector with 512 dimensions, the attention weights are computed using two fully connected layers followed by a softmax function:


$$\text{A}=\text{Softmax}\left({\text{W}}_{2}\cdot \text{Tanh}\left({\text{W}}_{1}{\text{H}}^{T}+{\text{b}}_{1}\right)+{\text{b}}_{2}\right)$$

where ${\text{W}}_{1}\in {\mathbb{R}}^{512\times 128}$ and ${\text{W}}_{2}\in {\mathbb{R}}^{128\times 1}$ are learnable weight matrices, and ${\text{b}}_{1}\in {\mathbb{R}}^{128}$ and ${\text{b}}_{2}\in {\mathbb{R}}^{128}$ are bias terms. The attention weights of each instance can be simplified as:


$${\text{a}}_{\text{i}}=\frac{\text{exp}\left({\text{W}}_{2}^{\text{T}}\cdot \text{Tanh}\left({\text{W}}_{1}{\text{h}}_{\text{i}}+{\text{b}}_{1}\right)+{\text{b}}_{2}\right))}{\sum_{\text{j}=1}^{\text{N}}\text{exp}\left({\text{W}}_{2}^{\text{T}}\cdot \text{Tanh}\left({\text{W}}_{1}{\text{h}}_{\text{j}}+{\text{b}}_{1}\right)+{\text{b}}_{2}\right)}$$

This approach automatically assigns higher weights to important instances, thereby improving overall discriminative power. The weighted feature vector M is then obtained by:


$$\text{M}=\sum_{\text{i}=1}^{\text{N}}{\text{a}}_{\text{i}}{\text{h}}_{\text{i}}$$

Eventually, the aggregated feature vectors are passed through the fully connected layer and dichotomized (benign or malignant) using the sigmoid activation function.

### 2.2 Experiment

#### 2.2.1 Datasets

The thyroid ultrasound image data used in this study were obtained from the Affiliated Hospital of Hangzhou Normal University, and the images were acquired using ultrasound equipment (Mindray DC-8 and Mindray Resona 7EXP) with different numbers of ultrasound images obtained in a single examination. A total of 2000 patients were enrolled between January 2018 and July 2024. Ultrasound reports were collected for each examination. According to the C-TIRADS diagnostic criteria (9), the dataset includes 1000 patients with category 3 nodules, 975 patients with category 4 nodules, and 25 patients with category 5 nodules. Nodules classified as C-TIRADS category 3 were considered low-risk, while those in categories 4 and 5 were considered high-risk. A total of 16229 ultrasound images from 2000 patients with thyroid nodules were collected for this study. Of these, 12,283 images from 1500 patients were used for the training set, 750 patients had low-risk nodules and 750 patients had high-risk nodules. The test set consisted of 3946 ultrasound images from 500 patients, of which 250 patients were low-risk nodules and 250 patients were high-risk nodules. The mean age of the patients in the training and test sets was (49.83 ± 11.72) and (46.23 ± 11.46) years, respectively. The demographic characteristics of the two data sets are shown in **Table 1**.

**Table 1:** Demographic data for two data sets.

Parameter	Total	Training set	Test set
Patients	2000	1500	500
Male, n (%)	1008 (50.4%)	780 (52%)	272 (54.4%)
Female, n (%)	992 (49.6%)	720 (48%)	228 (45.6%)
Age (years)	48.93 ± 11.76	49.83 ± 11.72	46.23 ± 11.46
Total images	16229	12283	3946
low-risk nodules patients, n (%)	1000	750 (50%)	250 (50%)
high-risk nodules patients, n (%)	1000	750 (50%)	250 (50%)

In this study, all images from a single examination shared the same label, indicating that the examination had a high-risk nodule. All examinations were classified into two categories: high-risk and low-risk for thyroid nodules and were labeled according to the ultrasound report. The labeling process was performed by a single sonographer. The study was conducted in accordance with the Helsinki Declaration, and approved by the Ethics Committee of our institution (Approval number: 2024 (E2) -KS-160).

#### 2.2.2 Data preprocessing

The dataset was randomly divided into a training set and a test set at a ratio of 3:1, where 75% of the data were used for training the model and 25% of the data were used for the final evaluation of the model. The entire dataset division process ensured that different patient categories were distributed in both training and test sets. All the images were preprocessed uniformly before being fed into the model to ensure stability and robustness. First, the images were resized to 512 × 512 pixels. To enhance the generalization ability of the model, the images were enhanced using random flipping and contrast enhancement. These transformations simulate different imaging conditions and increase the robustness of the model. Finally, the images were normalized to the pixel values.

#### 2.2.3 Training strategies

The model was trained using the Adam optimizer with an initial learning rate of 0.00005 and a batch size of 1 (one patient per batch). The loss function was chosen as the Binary Cross-Entropy (BCE). NVIDIA RTX3090 was used for model training. The entire training process was conducted in an end-to-end manner, taking approximately 12 hours to complete over 100 epochs.

## 3 Results

### 3.1 Assessment of indicators

Several commonly used classification evaluation indices were used in this study to comprehensively assess the performance of the model for thyroid risk diagnosis. The accuracy rate was used to measure the proportion of overall correct classifications of the model, and the precision indicated the proportion of actual positive cases when the model predicted a positive case, thereby reflecting the model’s ability to determine a positive class. On the other hand, recall measures the proportion of all actual positive cases correctly predicted by the model, reflecting the model’s ability to detect high-risk patients. The F1-Score serves as the reconciled mean of precision and recall, which combines the precision and recall of the model. In addition, we plotted receiver operating characteristic (ROC) curves to evaluate the model’s ability to discriminate between dichotomous classification tasks under different thresholds. The higher area under the curve (AUC) value, the better is the overall classification ability of the model. Together, these evaluation metrics measure the performance of the model in thyroid nodules risk diagnosis and validate its accuracy, reliability, and ability to detect high-risk cases from various perspectives. Additionally, we utilized Cohen’s d to quantify the effect size and assess the magnitude of differences between the proposed model and comparison methods. Cohen’s d is a measure of the standardized difference between two means. It provides insight into the practical significance of the model’s improvements. In this study, a Cohen’s d value around 0.2 indicates a small effect size, around 0.5 represents a medium effect size, and around 0.8 corresponds to a large effect size. This effect size measurement helps to evaluate not only statistical significance but also the practical impact of the improvements made by our model.

### 3.2 Analysis of feature extraction networks

In this experiment, we used several different convolutional neural networks as feature extractors and comparatively evaluated the performance of these networks in the risk diagnosis of thyroid nodule ultrasound images under an attention-based multi-instance learning framework. The selected networks include the ResNet family, AlexNet, the VGGNet family, the MobileNet family, and the DenseNet family, which represent typical current deep learning models of different complexity and structural design.

As can be observed from the results in **Table 2**, VGG13 performed well on several evaluation metrics and became the best feature extraction network for this task. Specifically, VGG13 significantly outperforms the other networks in accuracy (0.8386), precision (0.8512), recall (0.8427), and F1-Score (0.8380), demonstrating the advantages of its deep convolutional structure in capturing the features of ultrasound images of the thyroid nodule. Notably, the comparison between VGG13 and other models, such as ResNet34, AlexNet, VGG11, MobileNetv3-small, and DenseNet161, yielded statistically significant differences (p<0.0001). The performance of VGG16 is slightly inferior to that of VGG13, even though its F1 Score is closer in performance, but it is slightly inferior in precision and recall. This may be due to the fact that VGG16 has more convolutional layers, resulting in a model that is more prone to overfitting. In contrast, ResNet50, another deep residual network, performed well in terms of accuracy (0.7375), precision (0.7375), and recall (0.6875). The residual structure of ResNet helps avoid the problem of gradient vanishing in deep networks, making it a more stable performer for processing complex image data. However, the results also indicate that ResNet50’s performance is statistically inferior to VGG13 (p<0.0001). On the contrary, AlexNet performs poorly on all metrics, and its F1-Score is only 0.3623, which is much lower than that of the other models. This indicates that AlexNet’s shallow structure cannot effectively extract deep features in thyroid ultrasound images, resulting in its weak classification ability. The large effect size (Cohen’s d = 0.3903 compared to VGG13) underscores the substantial gap between these two models. The MobileNet series and DenseNet series perform in the middle of the list, especially MobileNet as a lightweight network, although its precision (0.6420) and recall (0.6416) are not outstanding, it has the limited computational resources still has some advantages. The DenseNet series performed well in terms of recall (0.6560) through the densely connected design of the feature graph, but the overall performance failed to outperform VGG13 and ResNet50. Although VGG13 demonstrates clear numerical advantages over ResNet18, VGG16, DenseNet169, and DenseNet201 across various metrics, it does not exhibit statistically significant differences. Furthermore, the Cohen’s d effect size is small (less than 0.02), indicating that, despite numerical differences, these variations may have minimal impact on patient predictions. However, due to the numerical superiority of VGG13, we still consider it to be a more suitable feature extraction network compared to the others.

**Table 2:** Results of feature extraction networks (VGG13 as the reference model, α=0.05 for statistical significance).

Feature extraction network		Accuracy	Precision	Recall	F1-Score	P-value	Cohen’s d
ResNet (34)	ResNet18	0.8169 ± 0.0353	0.8341 ± 0.0318	0.8218 ± 0.0321	0.8158 ± 0.0348	0.5642	0.0200
	ResNet34	0.5256 ± 0.0323	0.5920 ± 0.0302	0.5417 ± 0.0311	0.4600 ± 0.0337	< 0.0001	0.6734
	ResNet50	0.6772 ± 0.0342	0.7375 ± 0.0331	0.6875 ± 0.0335	0.6625 ± 0.0346	< 0.0001	0.3203
AlexNet (35)	AlexNet	0.4016 ± 0.0413	0.3777 ± 0.0489	0.4131 ± 0.0357	0.3623 ± 0.0392	< 0.0001	0.3903
VGGNet (36)	VGG11	0.7303 ± 0.0394	0.7741 ± 0.0350	0.7386 ± 0.0343	0.7233 ± 0.0396	< 0.0001	0.1886
	VGG13	0.8386 ± 0.0334	0.8512 ± 0.0301	0.8427 ± 0.0313	0.8380 ± 0.0344	/	/
	VGG16	0.7933 ± 0.0355	0.8073 ± 0.0338	0.7978 ± 0.0343	0.7923 ± 0.0364	0.4918	0.0240
MobileNet (37)	MobileNetv2	0.6398 ± 0.0429	0.6420 ± 0.0417	0.6416 ± 0.0416	0.6397 ± 0.0424	0.0347	0.1111
	MobileNetv3 small	0.7067 ± 0.0414	0.7134 ± 0.0434	0.7100 ± 0.0385	0.7061 ± 0.0426	0.0004	0.1717
DenseNet (38)	DenseNet121	0.6299 ± 0.0415	0.6291 ± 0.0410	0.6287 ± 0.0412	0.6288 ± 0.0416	< 0.0001	0.2461
	DenseNet161	0.6280 ± 0.0431	0.6277 ± 0.0427	0.6248 ± 0.0415	0.6242 ± 0.0418	< 0.0001	0.3391
	DenseNet169	0.6516 ± 0.0413	0.6614 ± 0.0416	0.6560 ± 0.0407	0.6497 ± 0.0425	0.8145	0.0120
	DenseNet201	0.6575 ± 0.0418	0.6694 ± 0.0415	0.6623 ± 0.0402	0.6551 ± 0.0420	0.5302	0.0321

**Figure 1:**
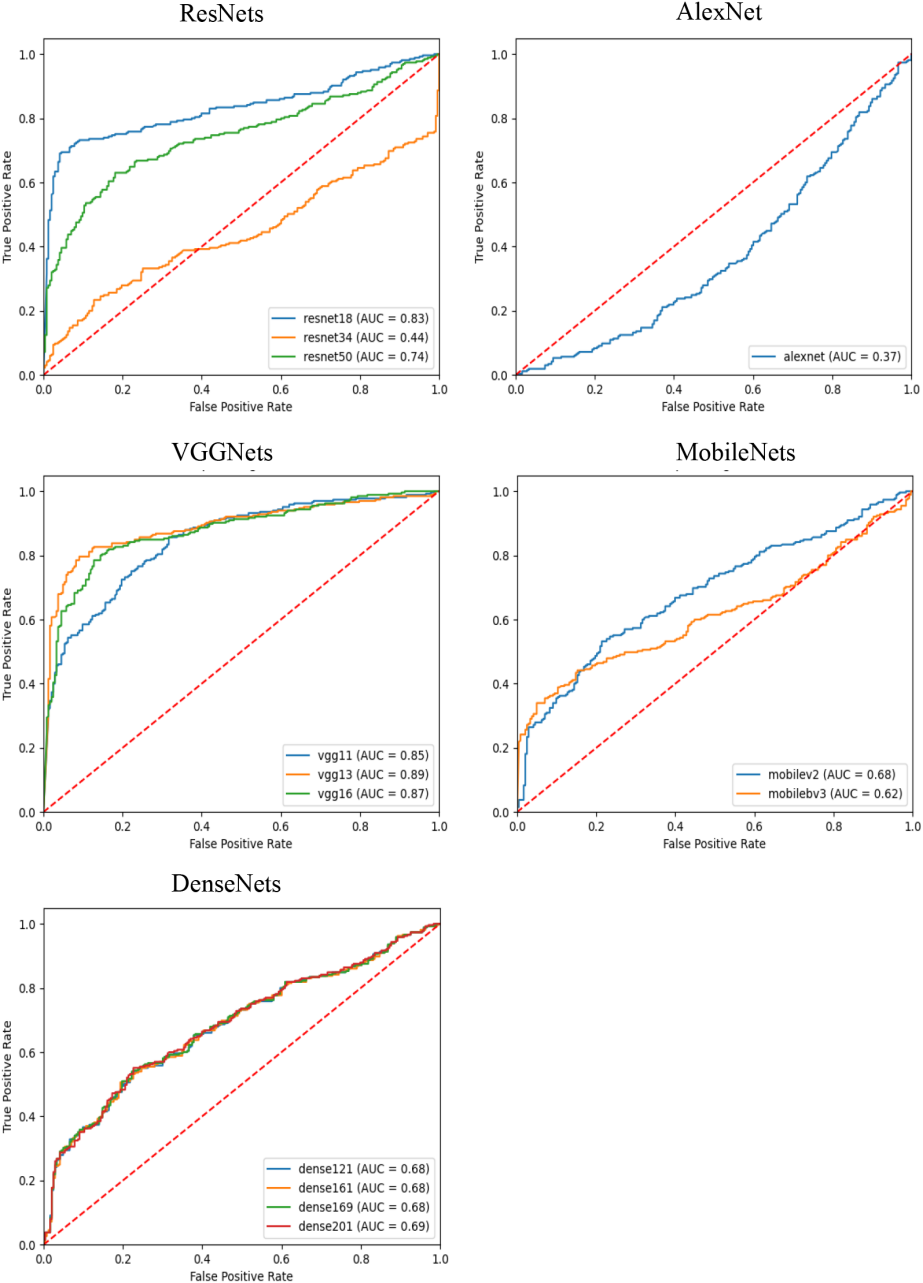
ROC curves of compared methods on the test set.

As shown in **Figure 1**, the AUC-ROC curves further demonstrate the performance of each model under different classification thresholds. We measured the comprehensive classification ability of the models using the AUC value, and the results showed that VGG13 had the highest AUC value of 0.8900, which further proved the excellent performance of VGG13 in thyroid nodules ultrasound image feature extraction.

### 3.3 Comparative with SOTA networks

To further validate the effectiveness of the proposed model, we designed an experiment to compare its performance with other models commonly used for thyroid nodules ultrasound image classification. The selected comparison models included LoGoNet (22) and ThyNet (23), both which are currently popular thyroid nodules classification models. All the models were trained on the same dataset and evaluated using the same metrics.

As can be seen from the experimental results in **Table 3**, LoGoNet has accuracy (0.7839) and precision (0.9267), but its low recall (0.6497) suggests that it may miss some malignant cases. The combination of high precision and low recall may reflect the model’s superior performance in avoiding misdiagnosis of benign cases but at the cost of overlooking high-risk patients. In contrast, ThyNet has a better recall rate than LoGoNet, but its precision rate is slightly lower, suggesting that ThyNet performs better in capturing malignant cases but also has a higher false positive rate. Overall, ThyNet strikes a balance between recall and precision but is still inferior to our proposed model.

**Table 3:** Results of our model and comparison methods. (VGG13 as the reference model, α=0.05 for statistical significance).

Method	Accuracy	Precision	Recall	F1-Score	AUC	p	Cohen’s d
LoGoNet	0.7839 ± 0.0267	0.9267 ± 0.0235	0.6987 ± 0.0249	0.7967 ± 0.0251	0.6340 ± 0.0200	< 0.0001	0.1824
ThyNet	0.7999 ± 0.0188	0.9039 ± 0.0154	0.7497 ± 0.0163	0.8196 ± 0.0178	0.8851 ± 0.0124	< 0.0001	0.3762
Ours	0.8386 ± 0.0334	0.8512 ± 0.0301	0.8427 ± 0.0313	0.8380 ± 0.0344	0.8900 ± 0.0309	/	/

Our proposed multi-instance learning model based on VGG13 performs well on several metrics, especially with significant advantages in recall (0.8427) and AUC (0.8900). This indicates that the model is more reliable for identifying high-risk thyroid nodules patients and effectively reduces the leakage rate. Recall, which measures the proportion of all actual positive cases that are correctly predicted as positive by the model, is particularly important for thyroid nodules classification. A higher recall means that the model can capture more malignant cases, thus reducing the risk of underdiagnosis. The experimental results show that the recalls of LoGoNet, ThyNet, and our model were 0.6497, 0.7497, and 0.8427, respectively. Our proposed model significantly outperformed the other two comparative models in terms of recall, indicating that it is more reliable in detecting high-risk patients and reduces the likelihood of missed diagnosis. Thus, our model has important implications for clinical diagnosis and is particularly superior in avoiding missed diagnoses, which may pose serious health risks.

However, a high recall usually leads to a decrease in precision because more positive class predictions may contain false positives. Therefore, whether the model has achieved a balance between precision and recall is a key question that we need to analyze further.The F1-Score is the reconciled mean of precision and recall, and combines the measures of how well the model is balanced on both metrics.The F1-Score is particularly important in unbalanced classification tasks, such as the thyroid cancer classification task, as it captures the model’s performance in identifying positive cases and avoiding false positives as a trade-off. The experimental results show that the F1-Score of LoGoNet, ThyNet, and our model are 0.7967, 0.8196, and 0.8380, respectively.Our proposed model also leads in F1-Score, indicating that the model achieves a better balance between precision and recall, which avoids a large number of false positives and is able to efficiently capture malignant cases.

In addition to the above metrics, statistical significance and effect size further validate the superiority of the proposed model. The p-values for both LoGoNet and ThyNet compared to our model are less than 0.0001, indicating that the differences are statistically significant. This highlights the reliability of our model’s improvements in classification performance. Cohen’s d, which measures the effect size, provides additional insights into the practical significance of these improvements. For LoGoNet and ThyNet, the Cohen’s d values are 0.1824 and 0.3762, respectively, suggesting small to medium effect sizes. This indicates that the improvements achieved by our model over ThyNet are more pronounced than those over LoGoNet.

Moreover, compared to SOTA networks, our model diagnoses at the patient level rather than on individual images, avoiding misdiagnoses due to poor image quality. Our attention mechanism operates across different images from the same patient, selecting critical diagnostic images and filtering out low-quality or misleading ones. These design choices enhance diagnostic reliability and improve model interpretability by highlighting clinically meaningful image segments.

In summary, the model proposed in this study demonstrates a comprehensive performance superior to that of existing models in the thyroid nodules ultrasound image classification task, especially in the identification and classification accuracy of high-risk patients, showing its great potential in clinical applications.

### 3.4 Analysis of attention mechanism

To evaluate the effectiveness of the attention mechanism in our MODEL, we conducted an ablation study comparing different instance aggregation strategies: max pooling, mean pooling, and our proposed attention-based aggregation. As shown in **Table 4**, the attention mechanism significantly outperformed both baseline pooling methods across all evaluation metrics. Specifically, our model achieved the highest accuracy.

**Table 4:** Ablation studies of attention mechanism.

Setting	Accuracy	Precision	Recall	F1-Score
Max pooling	0.8110 ± 0.0412	0.8261 ± 0.0425	0.8099 ± 0.0311	0.8110 ± 0.0376
Mean pooling	0.8012 ± 0.0405	0.8165 ± 0.0377	0.7999 ± 0.0451	0.8012 ± 0.0465
Ours	0.8386 ± 0.0334	0.8512 ± 0.0301	0.8427 ± 0.0313	0.8380 ± 0.0344

These results demonstrate that replacing simple pooling operations with an attention mechanism allows the model to adaptively assign different importance weights to each instance, thereby capturing more informative and discriminative features for patient-level classification. The superior performance confirms the critical role of attention in our MIL-based architecture.

### 3.5 Interpretable analysis

**Figure 2:**
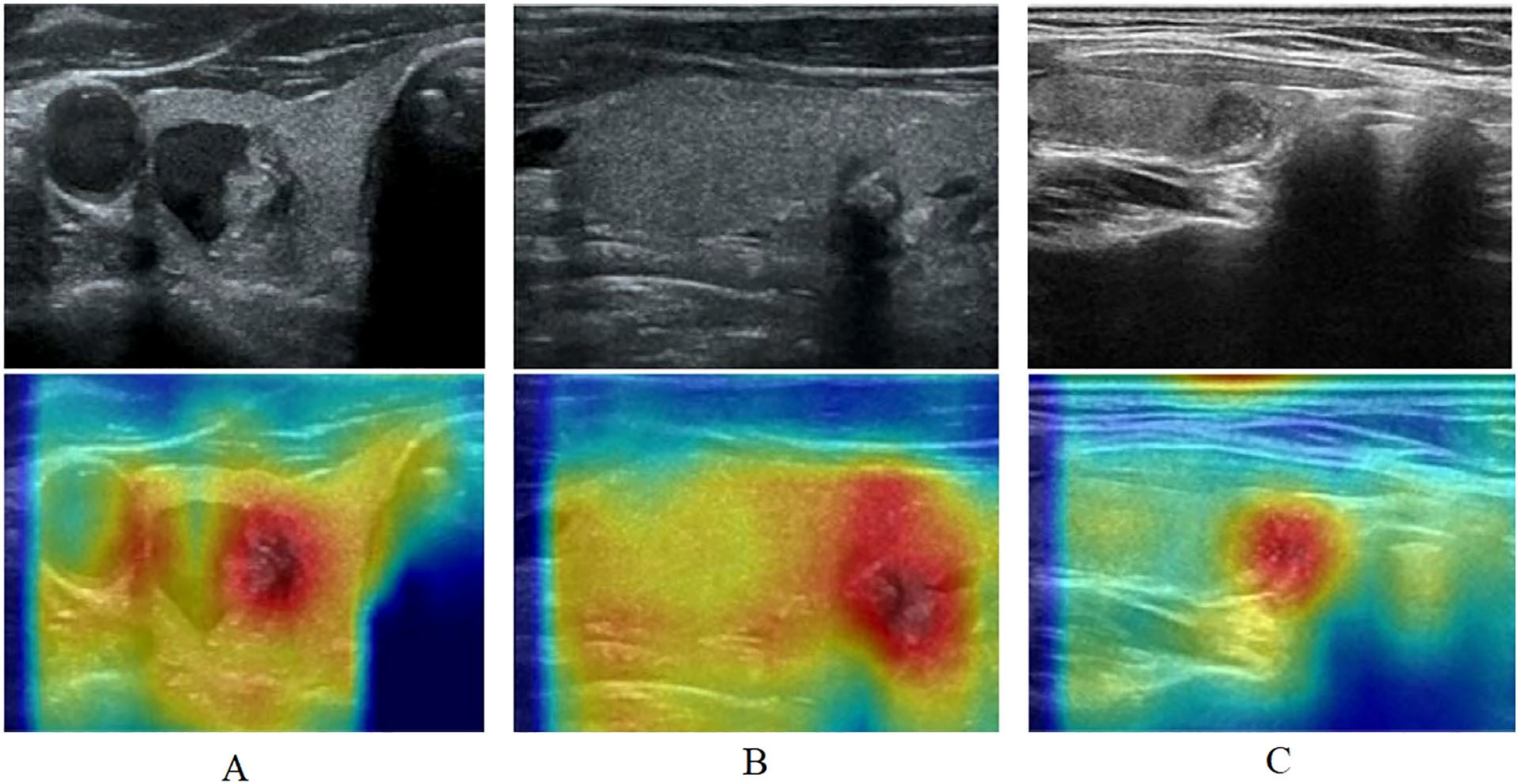
The heat map of attention for each ultrasound image using Grad-CAM. **(A)**: A includes ultrasonogram and heat map, ultrasonogram shows a mixed echogenic thyroid nodule, in which the solid portion has irregular morphology, unclear demarcation from the surrounding parenchyma, and multiple internal microcalcifications, diagnosed as a C-TIRADS category 4 nodule, heat map shows areas of dense aggregation with warm colors; **(B)**: B includes ultrasonogram and heat map, ultrasonogram shows an isoechoic thyroid nodule with irregular morphology, an aspect ratio of greater than 1, and posterior echogenic attenuation, diagnosed as a C-TIRADS category 4 nodule, heat map shows warm-toned color as an area of dense aggregation; **(C)**: C includes ultrasonogram and heat map, ultrasonogram shows a hypoechoic nodule with regular morphology and homogeneous internal echogenicity, diagnose as a C-TIRADS category 3 nodule, and heat map shows warm-toned color as an area.

To increase the interpretability of the model, we used Grad-CAM (39) to visualize and analyze the decision-making process of the model. Grad-CAM is a visualization technique that highlights the regions of an input image that most influence the model’s prediction, by leveraging the gradient information flowing into the final convolutional layer. Using Grad-CAM, a heat map of attention for each ultrasound image can be generated, as shown in **Figure 2**, thus revealing the key areas that the model focuses on when making classification decisions.

Based on the thyroid nodule ultrasound image features, the warm-toned region in the visualized image is the most important part that can be identified by Multi-instance learning convolutional neural network, and the darker color represents the higher risk of nodule classification. In the left column (A), ultrasonogram shows a mixed echogenic thyroid nodule, in which the solid portion has irregular morphology, unclear demarcation from the surrounding parenchyma, and multiple internal microcalcifications, diagnosed as a C-TIRADS category 4 nodule, **Figure 2A** heat map shows areas of dense aggregation with warm colors; **Figure 2B** ultrasonogram shows an isoechoic thyroid nodule with irregular morphology, an aspect ratio of greater than 1, and posterior echogenic attenuation, diagnosed as a C-TIRADS category 4 nodule, **Figure 2B** heat map shows warm-toned color as an area of dense aggregation; **Figure 2C** ultrasonogram shows a hypoechoic nodule with regular morphology and homogeneous internal echogenicity, diagnosed as a C-TIRADS category 3 nodule, and **Figure 2C** heat map shows warm-toned color as an area.

### 3.5 Computational analysis

We further analyzed the computational costs of the compared methods by counting the averaging inference time of each patient. We listed the counted time in **Table 5**, where the results show that our model still retain outperforming computation efficiency when achieving the best accuracy. As our model only predict the probability once per patient rather than predict that once per image, the inference time of ours is lower than that of other models.

**Table 5:** Results of our model and comparison methods.

Method	LoGoNet	ThyNet	Ours
Inference Time (seconds per patient)	0.576	0.598	0.496

## 4 Discussion and conclusion

### 4.1 Discussion

This study developed a multi-instance learning convolutional neural network model of ultrasound features to automatically recognize thyroid nodules and perform risk assessment. Ultrasound images of 2000 patients with thyroid nodules diagnosed by sonographers based on C-TIRADS were collected. The results of this experiment show that the multi-instance learning convolutional neural network model outperforms several metrics, especially in recall (0.8427) and AUC (0.8900) as shown in **Table 2**. Our model is important for clinical diagnosis, especially for identifying and classifying high-risk patients with high accuracy, which can effectively reduce the risk of missed diagnoses.

In clinical applications, when physician encounters a thyroid nodule with uncertain or controversial C-TIRADS grading, we import multiple thyroid nodule ultrasound images into the multi-instance learning convolutional neural network model, which extracts more subtle features from the images, providing diagnostic information that cannot be obtained by the sonographer’s naked eye, and assisting the sonographer to make a more accurate judgment. In the future, we will also integrate the model into the ultrasound workstation, so that after the sonographer acquires multiple ultrasound images of thyroid nodules, the system automatically inputs the images into the model, and the model quickly outputs the prediction results of the nodule classification, providing real-time references for the physician to help him or her to more accurately perform the C-TIRADS grading. In the future, the model will further learn a large amount of multi-center clinical data to quantitatively assess the risk of malignancy of thyroid nodules and give a specific risk value to make up for the relative subjectivity of the C-TIRADS classification and provide physicians with a more accurate diagnosis basis.

Compared with previous studies, our study offers several advantages. First, while the MIL model has been widely used in the preprocessing stage of pathology images, we extended it to ultrasound images for thyroid nodule risk assessment. In this study, we considered the entire set of ultrasound image features of the patient, accounting for differences in lesion presentation across multiple views. By aggregating these features using the MIL framework, we achieved a more comprehensive and robust assessment of the patient’s overall lesion condition. The MIL approach enables the model to analyze the entire set of ultrasound images (i.e., video frames), rather than relying on a single image, which mitigates the risks of misjudgment and annotation inconsistencies. Additionally, MIL allows for patient-level assessments, where the risk label of the ultrasound image set is based on the physician’s diagnosis using C-TIRADS, eliminating the need to label each individual image. This significantly reduces labeling time and improves accuracy, offering advantages over traditional supervised learning models (16). **Table 2** compares our model with 13 typical deep learning models, including ResNet, AlexNet, VGGNet, MobileNet, and DenseNet, showing that our model achieves the best experimental results. In **Table 2**, comparisons with ResNet18, VGG16, DenseNet169, and DenseNet201 reveal no statistical differences between our model and these models. Moreover, VGG13 outperforms the other models in accuracy (0.8386), precision (0.8512), recall (0.8427), and F1-Score (0.8380), demonstrating the advantage of its deep convolutional structure in capturing thyroid ultrasound features. The highest F1-Score indicates a good balance between precision and recall, minimizing false positives while capturing malignant cases effectively. Statistical analysis in **Table 2** supports the superiority of our model, showing significant differences compared to most models (p < 0.05). For instance, compared with ResNet34, ResNet50, AlexNet, and DenseNet161, our model not only exhibits statistically significant differences but also demonstrates substantial improvements with large effect sizes (Cohen’s d > 0.3). Additionally, the differences between our model and VGG11, the MobileNet series, and DenseNet121 are statistically significant (p < 0.0001). However, their effect sizes are smaller (Cohen’s d < 0.2), indicating moderate to minor practical differences. In cases where statistically non-significant results were observed, they may still hold value in the broader context of this study. For example, when comparing our model with ResNet18, VGG16, DenseNet169, and DenseNet201, although no statistically significant differences were identified, the observed performance metrics consistently favor our model. This consistency suggests that our approach is at least comparable, if not superior, in these scenarios. Moreover, the lack of statistical significance could be attributed to the relatively small effect sizes (Cohen’s d < 0.2) or the inherent variability within the dataset, rather than a true lack of difference. When compared with the baseline methods, our model demonstrated statistical significance, with effect sizes of 0.3762 and 0.1824, respectively. Our model has satisfactory classification performance, is more reliable in identifying high-risk thyroid patients, effectively reduces the leakage rate, and is important in clinical diagnosis, helping improve physicians’ ability to recognize the benign and malignant nature of thyroid nodules.

The model provides physicians with objective risk assessments by extracting aggregated features from thyroid nodule ultrasound images. This helps doctors make more informed treatment decisions, combining these results with clinical history and other test outcomes. Accurate risk assessment is crucial for determining appropriate treatment options. The model reduces unnecessary invasive tests and treatments while ensuring timely intervention for high-risk nodules. By automating feature extraction and risk assessment, the model enhances the efficiency of medical treatment, particularly in areas with a high prevalence of thyroid nodules, and significantly improves the accuracy and efficiency of ultrasound examinations.

### 4.2 Limitations

This study has several limitations. First, demographic information was not collected at the time of data collection for deeper sensitivity analysis. In future studies, baseline information on patients and populations receiving FNA for thyroid nodules should be collected to increase the diversity of available data. Second, single data modality was used in this study. The absence of such multi-modal data limits the model’s ability to provide a comprehensive risk assessment. Integrating these additional data sources in future studies could enhance diagnostic accuracy and personalization. Third, all data used in this study were collected from a single medical center, raising concerns about the generalizability of our findings. Variations in imaging protocols, equipment, and patient populations across institutions may affect model performance. To address this limitation, we plan to conduct cross-center validation and external testing in future work to evaluate the model’s robustness and adaptability in diverse clinical environments. Fourth, obtaining large-scale, high-quality labeled ultrasound datasets remains a significant challenge. Manual annotation by expert radiologists is time-consuming, costly, and subject to inter-observer variability. These factors can limit the scalability and consistency of training data. Future research should explore weakly supervised or semi-supervised learning approaches to reduce reliance on extensive manual labeling. Fifth, data annotation was performed by a single expert radiologist, which may introduce labeling bias. Future work should include multiple annotatorsm and consensus-based labeling for ambiguous cases. Although this study shows potential for assessing the risk of thyroid nodules, further research and improvements are needed to increase its validity and reliability in practical clinical applications.

### 4.3 Conclusion

In conclusion, the proposed multi-instance learning convolutional neural network model enables accurate and objective identification of high-risk thyroid nodules using multiple ultrasound images. Unlike traditional methods that rely heavily on subjective interpretation, our model leverages deep learning and attention mechanisms to automatically classify, localize, and assess the malignancy risk of thyroid nodules. To support this study, we constructed a real thyroid ultrasound dataset and performed a comprehensive comparison with existing thyroid nodule classification models using ultrasound images. The experimental results show that our convolutional classification model based on multi-instance learning exhibits an excellent performance. In summary, our innovative approach not only provides a new direction for expanding the application of multi-instance learning in whole-slice images (WSI) but also provides an automated C-TIRADS-based solution for thyroid nodules risk assessment.

## Data Availability

The raw data supporting the conclusions of this article will be made available by the authors, without undue reservation.
